# Clerodane Diterpene Ameliorates Inflammatory Bowel Disease and Potentiates Cell Apoptosis of Colorectal Cancer

**DOI:** 10.3390/biom9120762

**Published:** 2019-11-21

**Authors:** Jia-Huei Zheng, Shian-Ren Lin, Feng-Jen Tseng, May-Jywan Tsai, Sheng-I Lue, Yi-Chen Chia, Mindar Woon, Yaw-Syan Fu, Ching-Feng Weng

**Affiliations:** 1 Department of Life Science and Institute of Biotechnology, National Dong Hwa University, Hualien 97401, Taiwan; jiahueizheng@gmail.com (J.-H.Z.); d9813003@gms.ndhu.edu.tw (S.-R.L.); fengjentseng@yeah.net (F.-J.T.); m655003@kmu.edu.tw (S.-I.L.); 2 Department of Orthopedics, Hualien Armed Force General Hospital, Hualien 97144, Taiwan; 3 Department of Neurosurgery, Neurological Institute, Taipei Veterans General Hospital, Taipei City 11217, Taiwan; mjtsai2@vghtpe.gov.tw; 4 Department of Physiology & Master’s Program, Kaohsiung Medical University, Kaohsiung 80708, Taiwan; 5 Department of Food Science & Technology, Tajen University, Pingtung 90741, Taiwan; ycchia@tajen.edu.tw; 6 Department of Radiation Oncology, Yeezen Hospital, Taoyuan 32645, Taiwan; woonmd@yahoo.com; 7 Department of Biomedical Science and Environmental Biology, Kaohsiung Medical University, Kaohsiung 80708, Taiwan; m805004@kmu.edu.tw; 8 Institute of Respiratory Disease, Department of Basic Medical Science, Xiamen Medical College, Xiamen 361023, China

**Keywords:** colorectal cancer, diterpenes, inflammatory bowel diseases, *Polyalthia longifolia*, herbal medicine

## Abstract

Inflammatory bowel disease (IBD) is general term for ulcerative colitis and Crohn’s disease, which is chronic intestinal and colorectal inflammation caused by microbial infiltration or immunocyte attack. IBD is not curable, and is highly susceptible to develop into colorectal cancer. Finding agents to alleviate these symptoms, as well as any progression of IBD, is a critical effort. This study evaluates the anti-inflammation and anti-tumor activity of 16-hydroxycleroda-3,13-dien-15,16-olide (HCD) in in vivo and in vitro assays. The result of an IBD mouse model induced using intraperitoneal chemical azoxymethane (AOM)/dextran sodium sulfate (DSS) injection showed that intraperitoneal HCD adminstration could ameliorate the inflammatory symptoms of IBD mice. In the in vitro assay, cytotoxic characteristics and retained signaling pathways of HCD treatment were analyzed by MTT assay, cell cycle analysis, and Western blotting. From cell viability determination, the IC_50_ of HCD in Caco-2 was significantly lower in 2.30 μM at 48 h when compared to 5-fluorouracil (5-FU) (66.79 μM). By cell cycle and Western blotting analysis, the cell death characteristics of HCD treatment in Caco-2 exhibited the involvement of extrinsic and intrinsic pathways in cell death, for which intrinsic apoptosis was predominantly activated via the reduction in growth factor signaling. These potential treatments against colon cancer demonstrate that HCD could provide a promising adjuvant as an alternative medicine in combating colorectal cancer and IBD.

## 1. Introduction

Colorectal cancer (CRC) has a significant health impact worldwide, and is a common cancer type in the United States. In addition, CRC is the third leading cause of cancer deaths for new cases, and second for estimated deaths in both genders in Taiwan [1]. In the majority of cases, it is a consequence of the progressive accumulation of genetic and epigenetic alterations that leads the transformation and progression of normal colorectal mucosa to adenoma and eventually carcinoma, progressing CRC [1]. When analyzed, the incidence of CRC includes 72% colon cancer and 28% rectum cancer [2]. Surgery is mostly the first choice for all stages of CRC treatment, while for stage IV of CRC, recurrent CRC, or liver cancer metastasis, chemotherapy is the main alternative strategy to treat CRC [3]. Clinically, chemotherapeutic drugs, including 5-fluorouracil (5-FU), oxaliplatin, and capecitabine, are commonly used to treat CRC; however, these typically contribute to several adverse effects, such as fatigue, nausea, bone marrow toxicity, immunosuppression, and easy bleeding. Moreover, chemotherapy is a conventional treatment for late-stage and recurrent colorectal cancer, bit the cancer cells frequently become drug-resistant after treatment [3], and the unpredictability of adverse or side effects ordinarily restricts the administration of an accurate dose. As incidences of adverse results are observed, a new discovery of more efficacious and less toxic agents against CRC is of great urgency.

Noticeably, the risk factors of CRC are complicated, and include excessive alcohol use, obesity, hereditary conditions, and long-standing inflammatory bowel disease (IBD) [3]. IBD is comprised of two conditions: ulcerative colitis (UC; ICD-10-CM code K51.90) and Crohn’s disease (CD; ICD-10-CM code K50.90), which are caused by uncontrolled gastrointestinal (GI) inflammation or bacterial infection that subsequently result in fever, edema, intestinal fibrosis, and ulcers [4]. Global prevalence rates for IBD exceed 0.3%, which is mostly attributed to human-development-index countries—e.g., the United States, Canada, Germany, Norway, and Australia—where incidence rates are twice as high as those of Asian and African countries [5]. Moreover, the incidence rates of IBD where it occurs in the industrialized countries of Asia, Africa, and South America were between 4% (Taiwan) and 11.1% (Brazil) from the 1990s to 2010 [5]. Therefore, IBD becomes a burden in Western and industrialized societies.

Within an inflamed GI tract, large numbers of immunocytes, including macrophages, T_H_ cells, neutrophils, and natural killer (NK) cells, are attracted and secrete various forms of cytokines, such as tumor necrosis factor alpha (TNF-α), interferon-γ (IFNγ), and interleukin (IL)-6. When the immunocytes are accumulated, cytokine secretions lead to reductions in incidences of inflammation, and finally potentiate carcinogenesis [6]. Furthermore, one study has illustrated the connection between IBD and the Wnt/β-catenin signaling pathways, which might trigger colon cancer progression and incidences of sporadic colon cancer [7]. Interestingly, vitamin D deficiency, genetic susceptibility, disturbance of the microbiome, and psychological status have recently been confirmed as risk factors for IBD [8,9]. Previous evidence has shown that chronic inflammation may cause GI tumorigenesis, which is familiar and lethal worldwide. Even the known risk factors of IBD that have been broadly explored, as well as the causes, complications, and medications of IBD, including CRC, are still unraveling [10]. Nonetheless, the treatment of IBD remains unsolved and needs to be explored. According to the inquiry of new drug discovery, 46% of new drugs are of natural origin, e.g., derivatives, precursors, or mimetic molecules [11]. This fact exemplifies the continuing research and development of new drugs from natural products, especially medicinal plants, to meet patient demand, as the prospect of exploring new compounds and alternative treatments gains an immediate importance.

*Polyalthia longifolia var.* pendula Linn. (family Annonaceae) is an ornamental tree originally distributed in India, Sri Lanka, and Pakistan, which also contains numerous biological functions, as presented in the literature [12]. By exploring bioactive components, a clerodane diterpene 16-hydroxycleroda-3,13-dien-15,16-olide (HCD; PubChem ID 124820) has been extensively identified [13]. In previous reports, HCD has been shown to have numerous medicinal values as an anti-inflammation, anti-cancer, anti-fungal, anti-diabetic, and anti-bacterial agent [14]. In our previous studies, HCD performed as an executor to induce autophagy in glioma cells and oral squamous cell carcinoma cells, which consequently triggered cancer cell death [15,16]. Moreover, HCD can induce anoikis and reduce cell proliferation via the regulation of both intracellular growth and focal adhesion signaling in renal carcinoma cells [17,18]. In addition to acting as an anti-tumor agent, HCD could also play a supplementary role in the cytotoxicity of tamoxifen-treated breast cancer via the modulation of the Bax/Bcl-2 ratio, which is directly expressed at cells undergoing apoptosis [19]. Recently, our studies have demonstrated the therapeutic potential of HCD against various types of cancers [19]. Nevertheless, the therapeutic potency of HCD in treating GI inflammation, e.g., IBD and colorectal cancer, has not been clarified. The aim of this study was to determine the dampening effect of HCD on IBD treatment and anticancer activity. In this work, two platforms containing an azoxymethane (AOM)/dextran sodium sulfate (DSS)-induced colitis IBD model (*in vivo*) and colorectal carcinoma cells Caco-2 (*in vitro*) were employed to evaluate the treated efficacy of HCD. Furthermore, the detailed mechanisms of HCD on anti-colorectal cancer were also investigated.

## 2. Experimental Section

### 2.1. Study Design

The main focus of this study was to evaluate the potential of HCD against IBD and colon cancer. The experimental design was divided into two parts: IBD induction and colon cancer with HCD treatment. In the IBD model, mice were induced by azoxymethane (AOM)/dextran sodium sulfate (DSS). HCD was intraperitoneally (i.p.) injected, and subsequently GI inflammation was observed. In the colon cancer part, Caco-2 cells were used as the sole platform for the determination of cytotoxicity and cell death characteristics, and the underlying mechanisms of colon cancer cytotoxicity in HCD-treated cells, including cell cycle signaling, growth factor signaling, and inflammatory signaling, were under investigation.

### 2.2. Chemicals

HCD was kindly provided by Professor Yi-Chen Chia (Department of Food Science and Technology, Tajen University, Taiwan). Isolation and identification of HCD has been described in the previous literature [20]. The reagents and mediums for cell culture were purchased from Thermo-Fisher (Waltham, MA, United States). General chemicals were obtained from Sigma-Aldrich (Merck KGaA, Darmstadt, Germany). The antibodies used in this study are listed in Table 1; these were purchased from Genetex International (Hsinchu, Taiwan), Cell Signaling Technology (Danvers, MA, United States), and Merck Millipore (Burlington, MA, United States).

### 2.3. In Vivo Test

#### 2.3.1. Animal Source and Care

Eight to ten-week-old C57BL/6 male mice were purchased from BioLASCO (Taipei, Taiwan) and kept in controlled environmental conditions (22 ± 2 °C, 55% ± 10% humidity, 12/12 h light/dark cycle). The animals were fed a commercial diet and water ad libitum. Mice experimental protocols were used according to the “Guide for the Care and Use of Laboratory Animals” of National Dong-Hwa University, approved by the National Dong-Hwa University Animal Ethics Committee (approval number 001/2016).

#### 2.3.2. Inflammatory Bowel Disease Model Induction and HCD Treatment

The in vivo IBD model was induced by the injection of AOM and DSS, following a previous study with slight modification [21]. At day 0, mice were weighed and i.p. injected with 10 mg/kg B. wt. of AOM. The next day, the mice were freely supplied with 2% DSS solution for an additional seven days, and regular water for a further seven days. This induction cycle was repeated once. The induction of IBD was verified by checking the histological change of the colon after mice were sacrificed at day 35.

Once IBD induction was confirmed, 15 mg/kg B. wt. of 5-FU, as well as 1.6 and 6.4 mg/kg B. wt. of HCD were i.p. injected once every three days until day 65. Mice were sacrificed by CO_2_ anesthesia, and their colons were collected for slicing in the literature [16].

### 2.4. In Vitro Test

#### 2.4.1. Cell Culture

Human colorectal carcinoma cell lines Caco-2 and HT-29 were obtained from the American Type Culture Collection (ATCC, Manassas, MA, United States). Caco-2 and HT-29 cells were cultured with Dulbecco’s modified Eagle medium (DMEM) or RPMI-1640, with 20% fetal bovine serum (FBS) and 1% penicillin/streptomycin (PS) supplementation, respectively. The environmental conditions were 37 °C and 5% CO_2_, and the cultured medium was renewed once every two days. Once cells reached 80% confluence, cells were detached by 0.25% trypsin/EDTA for further experiment. All experiments were carried out within 20 passes, with concern for uniformity and reproducibility.

#### 2.4.2. Cytotoxicity Assay

The cytotoxic effect of HCD was measured by MTT (3-(4, 5-dimethylthiazol-2-yl)-2, 5-diphenyltetrazolium bromide; MDBio Inc., Taipei City, Taiwan) assay, as previously described in the literature [16]. Briefly, 7 × 10^3^ cells per well of two cells were inoculated in 96-well plates before incubating with 0.5, 2.0, 5.0, and 10.0 μM of HCD and 1, 10, 50, and 100 μM of 5-FU (a positive control) for 36 and 48 h, respectively. The optical density (OD) at 570 nm was measured after incubation with MTT solution for 4 h, and was solubilized in DMSO. Cytotoxicity was presented by cell viability, which was the ratio of OD570 between treatment and untreated control (0 μM).

#### 2.4.3. Cell Cycle Analysis

The protocol of cell cycle analysis followed a previous study, with slight modification [16]. In brief, 7 × 10^4^ cells per well of Caco-2 cells were seeded into 12-well plates. Cells were incubated with 0.5, 2.0, and 5.0 μM of HCD for 36 and 48 h, respectively. Treated cells were fixed with 70% freeze ethanol and stained with propidium iodide (PI) at 37 °C for 1 h. The fluorescent intensity of PI within cells was detected by a Cytomics^TM^ FC 500 flow cytometer (Beckman-Coulter, Brea, CA, United States). Data from 10^4^ cells in each sample were collected, and the different cell cycles were analyzed.

#### 2.4.4. Western Blotting

A total of 2.5 × 10^5^ cells/well of Caco-2 cells were seeded into a six-well plate and incubated until 80% confluence. Cells were treated with 0.5, 1.0, and 2.0 μM of HCD for 24 and 36 h, respectively. After incubation, cells were homogenized, and the desired protein levels were analyzed according to the protocol described in a previous study [16]. The chemiluminometric intensity of each protein was normalized with GAPDH’s chemiluminometric intensity. The protein level change was represented by the ratio of normalized chemiluminometric intensity between treated and untreated groups.

### 2.5. Statistical Analysis

Data were expressed as mean ± SD from at least three independent experiments. The results were analyzed by one-way analysis of variance (ANOVA) with the Dunnett test. The significant difference (*p* < 0.05) was labelled “*” on the histogram produced by GraphPad Prism Ver 7.0 (GraphPad Software, La Jolla, CA, United States). The IC_50_ of the dose-dependent cytotoxicity was determined using non-linear regression embedded in GraphPad Prism, and the chosen model was the highest R^2^ value.

## 3. Results

### 3.1. Histological Change of Intestine Tissue after AOM/DSS Induction and HCD Treatment

**Figure 1:**
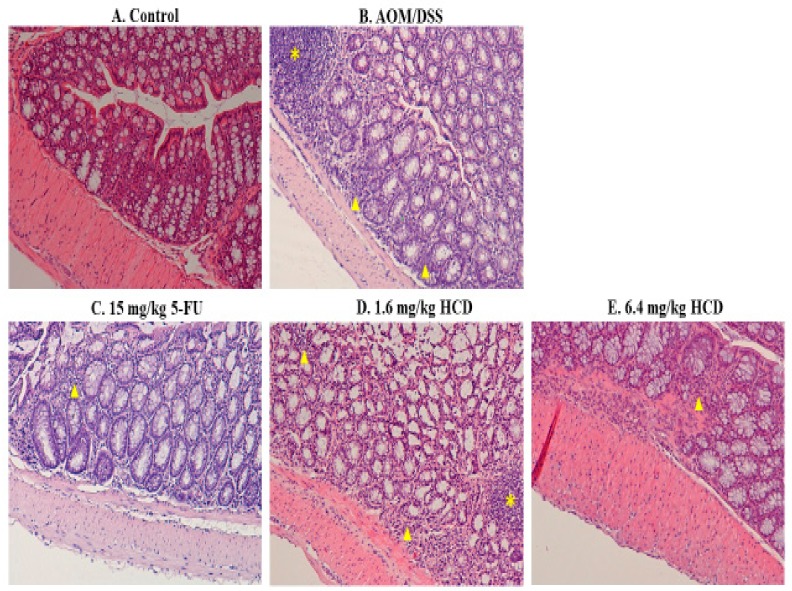
Histological appearances of the longitudinal section in the intestine of mice. Mice treated with (**A**) control and (**B**) azoxymethane (AOM)/dextran sodium sulfate (DSS) induction, as well as AOM/DSS induction followed by (**C**) 15 mg/kg B.wt of 5-fluorouracil (5-FU), (**D**) 1.6 mg/kg B.wt, and (**E**) 6.4 mg/kg B.wt of 16-hydroxycleroda-3,13-dien-15,16-olide (HCD) treatment (*n* = 5 in each group), were sacrificed, and the longitudinal section of tissues was stained with hematoxylin and eosin. The yellow asterisk and triangles indicate lymph node and lymphocyte infiltration, respectively, in the intestine. Magnification = ×100.

**Figure 2:**
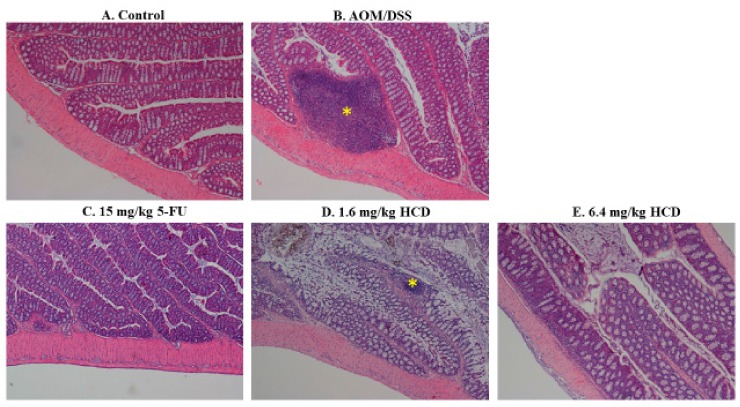
Histological changes of intestinal lymph nodes in HCD-treated mice with inflammatory bowel disease (IBD). The intestinal lymph nodes of (**A**) the control, (**B**) mice with AOM/DSS induction, and mice with AOM/DSS induction followed by (**C**) 15 mg/kg B.wt of 5-FU, (**D**) 1.6 mg/kg B.wt, and (**E**) 6.4 mg/kg B.wt of HCD treatment (*n* = 5 in each group) were stained and photographed. The yellow asterisk indicates the lymph node in the intestine. Magnification = ×40.

To generate the IBD mouse model, mice were chemically induced by AOM/DSS. After AOM/DSS induction, enlarged lymph nodes, lymphocyte infiltration, irregular and shorter villi, and thicker muscle mucous and muscle layers were observed in the intestines of mice, which consequently confirmed that mice were successfully induced with IBD after the AOM/DSS given (Figure 1B and Figure 2B), compared to the control without induction (Figure 1A and Figure 2A). In the next experiment, IBD-induced mice were employed to evaluate the amelioration efficacy of 5-FU and HCD on the histopathological signs of IBD. The tissue section showed that the lymphocytes were less or not infiltrated into the lamina propria layer after treatment with 5-FU and HCD (Figure 1C–E). The arranged villi in neat rows were found in an induced group as a positive control (AOM/DSS alone), and this feature was not observed in the 5-FU and HCD-treated groups. Additionally, the lymph nodes were reduced after treating with 5-FU and HCD (Figure 2C–E). These histological changes elicited that HCD could reduce IBD symptoms. The next experiments were performed to evaluate the efficacy of HCD on colorectal cancer cells.

### 3.2. Cytotoxicity Effects of HCD and 5-Fluorouracil on Colorectal Cancer Cells

**Figure 3:**
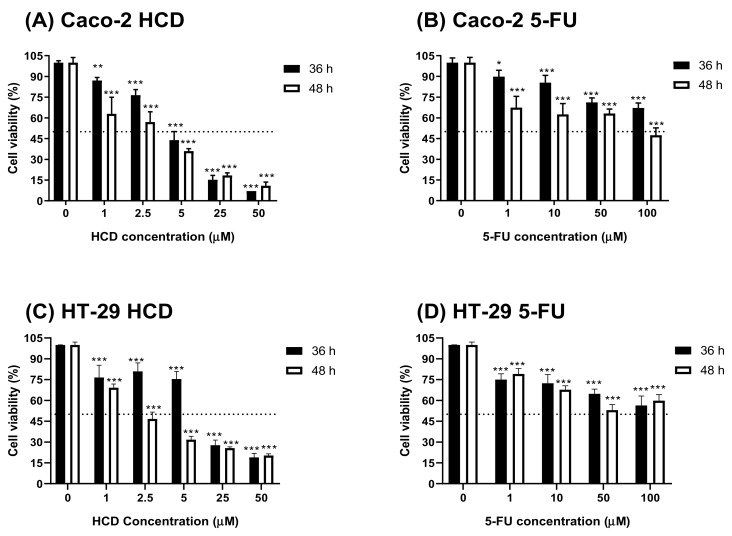
Cell viability of Caco-2 and HT-29 cells after HCD and 5-FU treatments. HCD and 5-FU were used to treat with (**A**,**B**) Caco-2 and (**C**,**D**) HT-29 cells for 36 and 48 h, respectively. The cell viability was determined by MTT assay. In addition, the cell viability was calculated according the comparison of control group (0 μM). Data (*n* = 3) were represented as mean ± SD. * *p* < 0.05; ** *p* < 0.01; *** *p* < 0.001, as compared with the untreated control (0 μM).

To check cytotoxicity of HCD against colorectal cancer cells, cells were treated with various concentrations of HCD and 5-FU (conventional chemotherapeutic agent, as a positive control), respectively. When compared to the untreated control (0 μM), cell viability in HCD-treated groups was significantly decreased (Figure 3A). The IC_50_ values of HCD in Caco-2 cells were 4.10 μM (36 h) and 2.32 μM (48 h), which were lower in 5-FU (100 μM for 36 h; 66.79 μM for 48 h) (Figure 3B). To further validate the potential of HCD for colon cancer treatment, another colon cancer cell, HT-29, was treated with various concentrations of HCD. The results showed that a dose-dependent decrease of cell viability was also observed in HCD-treated HT-29 cells. The IC_50_ values of HCD against HT-29 were 10.18 μM (36 h) and 1.39 μM (48 h), and were higher than those of Caco-2 (Figure 3C). According to these results, we confirmed that the cytotoxicity of HCD in colorectal cancer cells (Caco-2 and HT-29) had a higher potential than 5-FU. Therefore, the subsequent experiments were focused on the investigation of underlying mechanisms in HCD against Caco-2 cells.

### 3.3. Characteristics of HCD-Induced Cell Death

**Figure 4:**
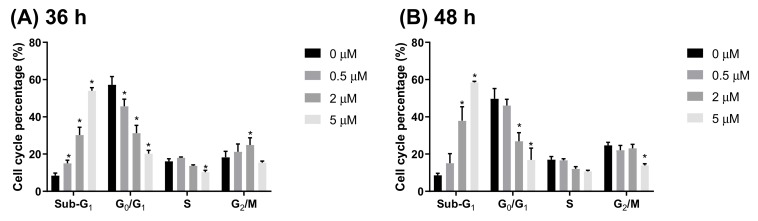
Cell cycle change after HCD treatment. Caco-2 cells were stained by propidium iodide after (**A**) 36 h and (**B**) 48 h of HCD treatment, and further analyzed by fluorescent intensity by flow cytometry. Data (*n* = 3) were represented as mean ± SD. * *p* < 0.05; ** *p* < 0.01; *** *p* < 0.001, as compared with the untreated control (0 μM).

**Figure 5:**
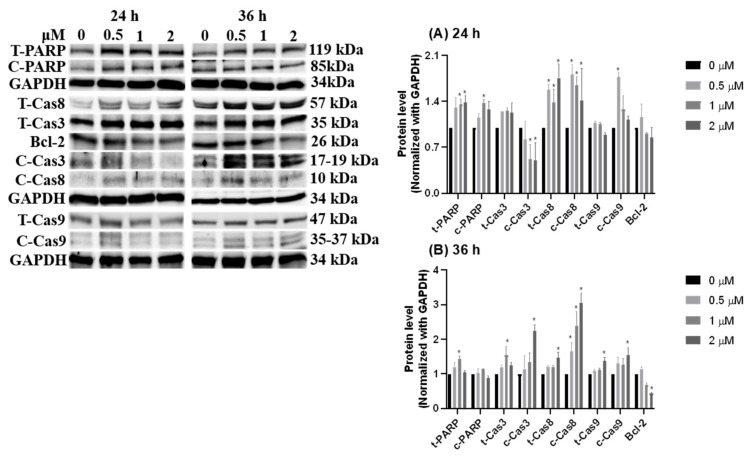
Dynamic change of apoptotic markers after HCD treatment. The protein levels of PARP, caspase-3, caspase-8, and caspase-9 were measured after (**A**) 24 h and (**B**) 36 h of HCD treatment. The protein levels were represented as the ratio of GAPDH-normalized chemiluminometric intensity between the untreated control (0 μM) and treatment. Data (*n* = 3) were represented as mean ± SD. * *p* < 0.05 as compared with the untreated control (0 μM).

To identify features of HCD-induced cell death, Caco-2 cells were treated with various concentrations of HCD, and intracellular DNA content was checked using flow cytometry coupling with PI-staining. After 36 h and 48 h of HCD treatment, the sub-G_1_ and G_0_/G_1_ cell cycle exhibited significant change in a dose-dependent fashion (Figure 4A), which could be caused by increasing the sub-G_1_ ratio, referred to as the apoptotic population. Interestingly, the significant decrease of the G_2_/M phase was found only at 5 μM HCD (Figure 4B). Concurrently, analyzing apoptotic markers, the increase of cleaved caspase-3, -8, -9, and PARP specified that apoptotic cell death appeared at 24 h and 36 h of treatments, respectively (Figure 5). These results clearly illustrate that HCD-caused cell death in Caco-2 cells was dominate in apoptosis. Moreover, by observation of the dynamics of apoptotic markers, intrinsic apoptotic inducer caspase-3 expression was enhanced at the first 24 h, and then cleaved at the following 12 h (Figure 5A). Conversely, Bcl-2, the apoptotic inhibitor, had no significant change until 36 h of treatment (Figure 5B). Additionally, the extrinsic apoptotic markers, cleaved caspase-8 and -9, also had significantly higher expression at either 24 h or 36 h of treatment (Figure 5). The data confirmed that both intrinsic and extrinsic apoptotic signaling pathways were involved in HCD-induced apoptosis, and intrinsic apoptosis might be prior to the extrinsic event. The following experiments would be carried out to determine whether the dynamic change of growth signaling pathway is affected by HCD.

### 3.4. Growth Signal Reduced by HCD Triggered Caco-2 Cell Apoptosis 

**Figure 6:**
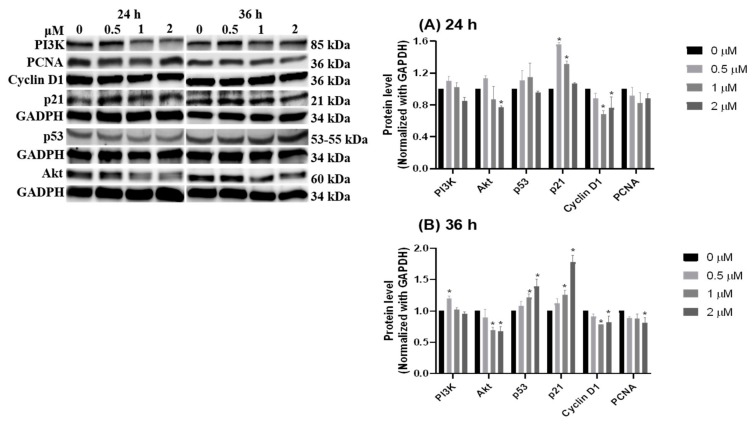
Dynamics of cell growth related signaling pathway after HCD treatment. Caco-2 cells were treated with HCD and the changes in PI3K, Akt, p53, p21, cyclin D1, and PCNA were measured at (**A**) 24 h and (**B**) 36 h. The protein levels were represented as the ratio of GAPDH-normalized chemiluminometric intensity between the untreated control (0 μM) and treatment. Data (*n* = 3) were represented as mean ± SD. * *p* < 0.05 as compared with the untreated control (0 μM).

Notably, the intrinsic apoptotic pathway is regulated by the balance of growth signal and anti-growth signal. The subsequent experiments were applied to measure the signals of PI3K/Akt for growth, p53/p21 for anti-growth, and cyclin D1/PCNA for cell division after HCD treatments. Western blot data indicated that the protein levels of Akt and cyclin D1 were significantly decreased in 2 μM of HCD treatment after the first 24 h, and a significant decrease of the cyclin D1 level at 1 μM of HCD treatment was observed (Figure 6A). Subsequently, the protein levels of p53 and p21 were up-regulated and PCNA was down-regulated at 36 h (Figure 6B). This result hinted that HCD-induced apoptosis could be the reducing result of growth signaling via Akt mediation. The downregulation of growth signaling caused the elevation of p53/p21 protein expression, and consequently turned down cyclin D1 expression, which was the key for overcoming the G1/S checkpoint. However, upstream of Akt, the protein levels of PI3K were not associated with the mediation of Akt (Figure 6), which meant that Akt might be reduced by other signaling pathways. The next section was performed to test the characteristics of the inflammatory pathway within HCD-induced apoptosis.

### 3.5. Inflammation-Suppressing Effect of HCD in Caco-2 Cells 

**Figure 7:**
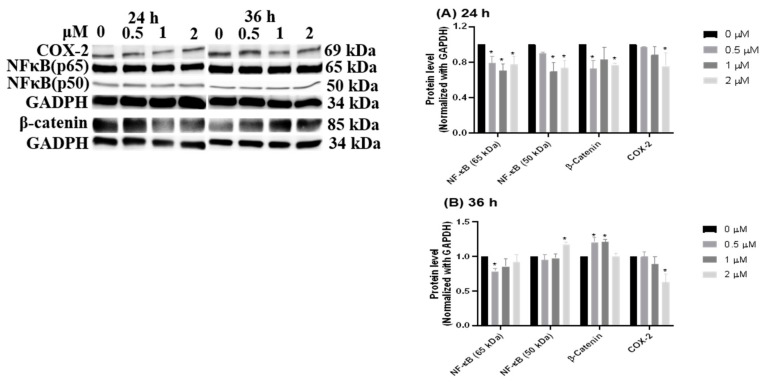
Inflammation-related protein changes after HCD treatments. NF-κB, β-catenin, and Cox-2 were determined by Western blotting at (**A**) 24 h and (**B**) 36 h HCD-treated Caco-2 cells. The chemiluminescent intensity of each protein was normalized with GAPDH and represented as the protein levels. Data (*n* = 3) were represented as mean ± SD. * *p* < 0.05 as compared with the untreated control (0 μM).

From the literature, inflammation has been proven as a promoting cause of colorectal cancer [22], and inflammatory signaling could crosstalk with Wnt/β-catenin, as well as being involved in colorectal cancer growth [23]. Thus, the subsequent experiment examined the regulation of inflammatory-related proteins, including COX-2, NF-κB, and β-catenin. At 24 h of HCD treatment, the p50 subunit of NF-κB showed a significantly decreasing manner (Figure 7A). The decrease of p50 was diminished, whereas β-catenin was up-regulated at 36 h (Figure 7B). This result refutes that NF-κB and β-catenin are involved in HCD-mediated apoptosis; however, the underlying mechanisms were not fully interpreted. Of note, inflammatory signaling was proven by the significant characteristics of IBD pathological progression, and then the subsequent experiment will be employed to evaluate whether oral gavage HCD may ameliorate IBD symptom in an AOM/DSS- induced mouse model by the mediation of inflammatory signals.

## 4. Discussion

In chemical-induced IBD and colitis-associated cancer research, this study broadened the application of HCD in medical use, and could provide a new approach for IBD treatments. These experimental results conferred the anticancer effect of HCD against colon cancer, which led to intrinsic and extrinsic regulation for apoptotic cell death by down-regulating Akt-mediated growth signaling. Moreover, the anti-inflammation fashion of HCD in colon cancer might be one reason for the down-regulation of Akt, and is postulated for the IBD curing effect in vivo.

Usually, the conventional therapies for IBD can be grouped into the two following approaches: anti-inflammation, *e.g.*, corticosteroids, mesalazine, and cyclosporine; and anti-microbial, including ornidazole and rifaximin [24]. Ordinarily, these drugs are useful for treating mild to moderate IBD. However, side effects, such as drug resistance to antibiotics and opportunistic infection, are still of concern [25]. Likewise, about one-third of chronic IBD patients have failed responses to corticosteroid medication, which are valid for acute IBD [25]. Hence, natural components are potentially considered to be the new strategies or approaches for curing IBD. Five types of phenylpropanoids and four types of lignan glycosides—phytochemicals originated from a warm-season perennial legume, *Lespedeza cuneata*—were synthesized, and these compounds could ameliorate UC [26]. Curcumin, the primary active compound of turmeric, alleviates CD and UC by inhibiting NF-κB activity [27]. Macrophage infiltration into the intestines could also be impaired by α-eleostearic acid isolated from *Momordica charantia* [28]. After AOM/DSS induction, polypus and enlargement lymph nodes were found in the colon by histopathological examinations in our study (data not shown). Additionally, the observations of irregular villi arrangement and lymphocyte infiltration into the lamina propia layer in this study are typical characteristics of IBD. The IBD mice treated with HCD had neat rows of villi and lymph nodes that were not enlarged or infiltrated into lamina propia layer, which indicates that HCD could relieve the symptoms of IBD.

In indigenous medication, *P. longifolia* is an antipyretic drug used in past decades [12]. Under modern chemical and medical research, various antimicrobial and anti-inflammatory compounds are purified and identified, such as 16α-hydroxy-cleroda-3,13(14)-Z-diene-15,16-olide, (3S,4R)-3,4,5-trihydroxy pentanoic acid-1,4-lactone and 16-Oxo-cleroda-3,13E-dien-15-oic acid [12]. HCD has been demonstrated to alleviate lipopolysaccharide-induced microglia inflammation via reducing iNOS, COX-2, and NF-κB gene expression [13]. In addition, HCD repressed COX-2 protein expression in Caco-2 and further ameliorated inflammation in AOM/DSS-induced mice. These results imply that HCD represents a novel and potential clinical approach for IBD treatment and CRC chemotherapy.

In our previous study, HCD was demonstrated to be a non-toxic agent to normal cells [16]. When compared to 5-FU, the cytotoxicity of HCD against colorectal cancer was higher than 5-FU (4.10 vs. 100 μM in IC_50_ at 36 h treatment), suggesting that HCD had higher efficacy and more potent when applied to colon cancer treatments. In the cell cycle analysis of Caco-2 cells treated with HCD, the ratio of the sub-G_1_ phase was significantly increased, and this increase was associated with an increase of HCD concentrations. This result was accompanied by the analysis of pro-apoptotic markers, such as PARP; caspase-3, -8, and -9; and Bcl-2. During observations from 24 h to 36 h of HCD treatment, the protein levels of pro-apoptotic molecules changed, and these signaling transductions could be precisely determined by caspase-8, caspase-3, Bcl-2, and PARP. Again, the caspase-3 expression level was increased in the first 24 h, which also indicated that intrinsic apoptotic pathways were activated at this time. These results imply that both intrinsic and extrinsic apoptotic signaling pathways were simulanteously activated in Caco-2 cells. This was the first evidence that HCD could induce the apoptosis of cancer cells.

As we know, extrinsic and intrinsic apoptotic signaling pathways exert themselves with different signaling molecules [29]. In general, an extrinsic apoptotic signaling pathway is started from the activation of death receptor (TRAIL receptor or TNF receptor) and terminated at pro-caspase-3 cleavage via caspase-8 activation [29,30]. Different from extrinsic apoptosis, intrinsic apoptotic signaling is mediated by Bcl-2 to cause the loss of mitochondrial membrane potential or growth signaling depletion, which leads to cytochrome c release, pro-caspase-9 cleavage, and consequently, caspase-3 activation [29,31,32]. Therefore, by observing the altered levels of cleavage caspase-8, caspase-9, and Bcl-2, the type of chemical-induced apoptosis could be putatively addressed. Previous studies of HCD-induced cancer cell death were focused on autophagic cell death and intrinsic apoptosis [15,16,33,34]. To the best of our knowledge, this study is the first to show the involvement of HCD-induced extrinsic apoptosis in colorectal cancer. Moreover, the underlined targets of HCD in extrinsic apoptosis still need to be further explored, because this work only focused on the alteration of caspase-8 proteins.

HCD-mediated intrinsic apoptosis was found by down-regulating β-catenin/NF-κB/Akt and activating p53/p21 expression. Previously, HCD had potentiated apoptosis via blocking the PI3K/Akt signaling pathway, promoting Aurora B degradation, and modifying histone-modifying enzymes in leukemia cells [33,34]. Remarkably, the cytotoxicity (cell death) of oral squamous cell carcinoma (OSCC) and glioma cells were also demonstrated in the treatment of HCD through an autophagic manner via Western blotting analysis, without an increase in sub-G_1_ [15,16]. Moreover, HCD activated the autophagy in lung cancer (A549) via reducing the protein level of mTOR, PI3K/p85, Akt, and Beclin 1, and suppressed apoptosis by lessening cleaved-PARP formation (Chiu et al., 2019, unpublished data). Thereby, one possibility for cell death is that this difference in apoptosis or autophagy was regardless of different types of cancer cells (adenoma, carcinoma, glioma, or neuroblastoma) and p53 (wild or mutant type). The cell death features of HCD induced in Caco-2 cells and other cancer cells imply that the critical point of the apoptosis/autophagy switch needs to be clarified.

The known roles of p53 are in cell cycle regulation, apoptosis induction, DNA repair activation, and aerobic respiratory improvement [35]. When measuring the apoptosis-related protein levels, the p53 protein level showed an increase at 36 h after HCD treatment. In intestine and colon tissues under IBD, p53 was overexpressed by TNF-α induction, and subsequently triggered cell apoptosis [36]. Interestingly, the induction of TNF-α was only observed in mutant p53, but not in wild-type p53 [37]. Over 50% of colitis-induced colorectal cancer and colon neoplasia could be found with the TP53 mutation. This mutation is believed to be the first step of colitis-associated carcinogenesis when compared with sporadic CRC [38]. Of note, p53 in Caco-2 is an aberrant type [39]. HCD caused the alteration of p53 protein expression in CRC cells, which indicated that HCD might affect p53 protein levels in IBD tissues. Therefore, the effects of HCD on intestinal epithelial cells with wild-type p53 might differ from similar Caco-2 cells.

In addition, as an apoptosis-inducing feature, HCD also possesses inhibiting activity for Wnt/β-catenin in an anti-inflammation manner. Wnt/β-catenin dysregulation has been reported as a key factor for CRC initiation [40]. The Wnt/β-catenin signaling pathway acts as a central regulator in intestine homeostasis and epithelial stem cell proliferation. The Wnt ligand binds to the Frizzled/LRP receptor, and activates a signal cascade to subsequently result in the stabilization of β-catenin. Stable β-catenin can translocate into the nucleus and initiate gene expression of MYC and its downstream target, CCND1 [41]. Impaired activation of the Wnt/β-catenin signaling pathway could cause uncontrolled cell proliferation and finally, elicit colon cells carcinogenesis. Therefore, the Wnt/β-catenin signaling pathway would become the target of CRC prevention, prognosis, and diagnosis. Numerous studies have sought new compounds or herbal medicine for treating CRC. Fermented culture broth of *Antrodia camphorate*, hydnocarpin (a natural lignan), and bark extract of *Mesua ferrea* have been shown to inhibit activity of Wnt/β-catenin in colon cancer cells [42,43,44]. In this study, the reduction of β-catenin levels after HCD treatment showed the inhibited manner of the Wnt/β-catenin signaling pathway, which indicated a possibility of reducing colon cancer initiation.

## 5. Conclusions

This study illustrated anti-colorectal cancer activity from HCD by modifying intrinsic growth signaling and inflammatory modulators, which subsequently triggered both intrinsic and extrinsic signals to induce cell apoptosis. Furthermore, the inflammatory symptoms of AOM/DSS-induced enteritis in the in vivo mouse model were also ameliorated by HCD treatments. This is the first evidence showing the medicinal efficacy of HCD on IBD mice and colon cancer, suggesting that HCD could provide an alternative and complementary regimen for anti-colon cancer and IBD treatments.

## Figures and Tables

**Table 1:** Antibodies used in this study.

Protein Name	Molecular Weight (KDa)	Host	Manufacture	Dilution Factor
iNOS	131	Rabbit	Genetex	1:1000
PARP	119/85	+	Cell Signaling	+
PI3K	85	+	Genetex	+
β-catenin	85	+	+	+
COX-2	69	+	+	+
NF-κB (p65)	65	+	+	+
Akt	60	+	Cell Signaling	+
caspase-8	57/10	+	+	+
p53	53-55	+	+	+
NF-kB (p50)	50	+	Genetex	+
caspase-9	47/35-37	Mouse	Cell Signaling	+
WNT11	39	Rabbit	Genetex	+
cyclin D-	36	+	Cell Signaling	+
PCNA	36	Mouse	+	+
caspase-3	35/17-19	Rabbit	+	+
GADPH	34	+	Genetex	+
p27	27	+	Cell Signaling	+
Bcl-2	26	+	+	+
Bad	23	+	+	+
p21	21	+	Genetex	+
Bax	20	+	Cell Signaling	+
Anti-mouse HRP-conjugated 2nd Ab		Goat	Merck Millipore	1:5000
Anti-mouse HRP-conjugated 2nd Ab		+	+	+

+: Same as above.
